# Relaxin and Erythropoietin Significantly Reduce Uterine Tissue Damage during Experimental Ischemia–Reperfusion Injury

**DOI:** 10.3390/ijms23137120

**Published:** 2022-06-27

**Authors:** Lina Jakubauskiene, Matas Jakubauskas, Gintare Razanskiene, Bettina Leber, Jennifer Weber, Lisa Rohrhofer, Diana Ramasauskaite, Kestutis Strupas, Philipp Stiegler, Peter Schemmer

**Affiliations:** 1 General, Visceral and Transplant Surgery, Department of Surgery, Medical University of Graz, Auenbruggerplatz 2, 8036 Graz, Austria; morozovaite.lina@gmail.com (L.J.); matasjakub@gmail.com (M.J.); bettina.leber@medunigraz.at (B.L.); Jennifer.weber@medunigraz.at (J.W.); lisa.rohrhofer@medunigraz.at (L.R.); peter.schemmer@medunigraz.at (P.S.); 2 Faculty of Medicine, Vilnius University, M. K. Ciurlionio Str. 21, 03101 Vilnius, Lithuania; gintare.baranauskaite@vpc.lt (G.R.); diana.ramasauskaite@santa.lt (D.R.); kestutis.strupas@santa.lt (K.S.); 3 National Centre of Pathology, Affiliate of Vilnius University Hospital Santaros Klinikos, P. Baublio Str. 5, 08406 Vilnius, Lithuania

**Keywords:** transplantation, uterus, ischemia–reperfusion injury, relaxin, erythropoietin

## Abstract

Successful uterus transplantation, a potential treatment method for women suffering from absolute uterine infertility, is negatively affected by ischemia–reperfusion injury (IRI). The aim of this study is to investigate the protective effect of relaxin (RLX) or/and erythropoietin (EPO) on experimental uterus IRI. Eighty rats, randomly assigned into eight groups (*n* = 10/group), were pretreated with either saline, 5 μg/kg human relaxin-2, 4000 IU/kg recombinant human erythropoietin or their combination. Ischemia was achieved by clamping the aorta and ovarian arteries for 60 min, following 120 min of reperfusion and tissue sampling. For sham animals, clamping was omitted during surgery. There were no differences in tissue histological score, malondialdehyde (MDA) and superoxide dismutase (SOD) levels, myeloperoxidase (MPO) and TUNEL-positive cell count between all sham-operated rats. Pretreatment with RLX preserved normal tissue morphology, reduced MDA levels, MPO and TUNEL-positive cell count, preserved SOD activity and upregulated NICD and HES1 gene expression when compared to the control group. Pretreatment with EPO reduced MDA levels. In conclusion, pretreatment with RLX, EPO or a combination of both EPO and RLX significantly alleviates uterine tissue damage caused by IRI.

## 1. Introduction

Approximately 1.5 million women worldwide suffer from absolute uterine factor infertility and therefore have no chance to be both genetic and gestational mothers to their children [1]. Uterus transplantation (UTx) nowadays is considered to be the only treatment method for women who do not have a uterus due to congenital (uterus agenesis in Mayer–Rokitansky–Küster–Hauser syndrome) or acquired (hysterectomies due to large myomas, peripartum bleeding or sepsis and malignancies) causes [2,3]. Moreover, ethical, legal and technical issues of UTx are being discussed to implement this method for transgender individuals [4].

The first successful human UTx was performed in 2014 by a Swedish team led by Brännström [2]. Since then, the research interest in UTx has been rapidly growing as an increased number of surgical teams have already realized more than 40 successful UTx, including only a few utilizing uterine grafts from deceased donors, worldwide [5,6,7]. Organ procurement from a living donor enables the extensive evaluation of potential grafts before transplantation, including function, anatomy and the presence of vascular abnormalities [8]. On the other hand, living donors have a high risk of surgery-related complications, such as urinary tract and bowel injuries, bleeding and vaginal cuff dehiscence [1,2,9]. Considering the potential risks for living donors, research on the use of brain-dead donor organs is of growing interest. During organ donation, the uterus is retrieved last and therefore experiences longer warm ischemia times, resulting in reduced survival of the transplanted uterus [10].

Ischemia–reperfusion injury (IRI) plays a significant role during solid organ transplantation and can cause severe tissue alterations, leading to graft dysfunction and rejection [10]. The effect of IRI is mediated by several mechanisms, including overproduction of reactive oxygen species (ROS), neutrophil infiltration, endothelial dysfunction resulting in apoptosis and necrosis of cells in transplanted tissue [11]. Knowledge of the basic mechanisms underlying IRI allows the introduction of pharmacological adjuvants that could increase transplanted tissue tolerance to IRI [12].

Relaxin (RLX) is an insulin-related peptide hormone that has already demonstrated its antifibrotic, anti-inflammatory, antioxidative and cytoprotective effects during solid organ transplantation [13]. However, it has never been applied as a protective agent against IRI in a uterus model.

Erythropoietin (EPO) is an endogenous hormone that is already being used in clinical practice for treating anemia. Studies of hepatic and renal IRI models revealed that EPO can significantly alleviate IRI by reducing lipid peroxidation and serum tumor necrosis factor-α levels, increasing superoxide dismutase (SOD) activity and preserving tissue histology [14,15].

The aim of this study is to investigate the protective effect of RLX or/and EPO pretreatment on uterine tissue IRI in a rat model.

## 2. Results

### 2.1. Tissue Biochemical Analysis

**Figure 1:**
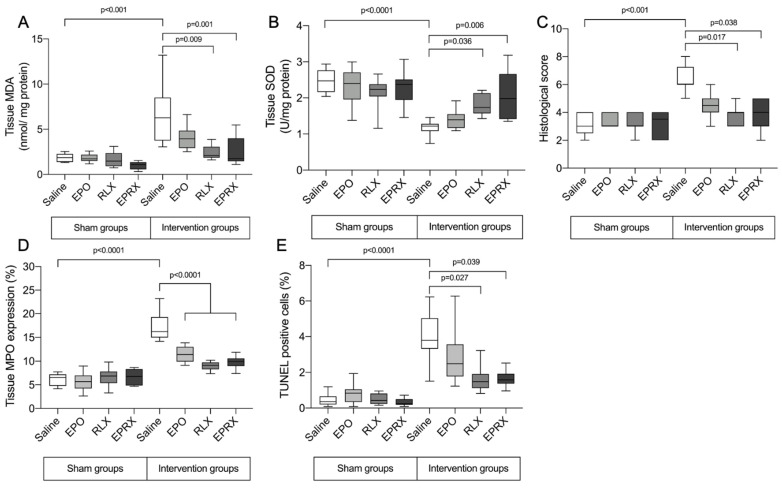
Uterine tissue lesion markers. MDA—malondialdehyde, SOD—superoxide dismutase, MPO—myeloperoxidase, TUNEL—terminal deoxynucleotidyl transferase-mediated dUTP nick-end labeling EPO—erythropoietin, RLX—relaxin, EPRX—erythropoietin and relaxin. Data presented as median (Q1; Q3). (**A**–**C**,**E**) Kruskal–Wallis test; (**D**) ANOVA test.

Malondialdehyde (MDA) and SOD concentrations were similar in all sham-operated groups (Figure 1). Uterine IRI led to significantly higher tissue MDA levels 2 h after reperfusion between the groups. The increase in tissue MDA levels was attenuated in groups pretreated with RLX and RLX with EPO compared to the saline-pretreated group. Additionally, SOD activity was significantly decreased in the saline-pretreated group (Figure 1). However, SOD activity remained higher in groups pretreated with RLX or RLX in combination with EPO compared to the control group. Pretreatment with EPO alone showed only a tendency to return MDA and SOD activity to normal.

### 2.2. Morphology, Neutrophil Infiltration and TUNEL Assay

**Figure 2:**
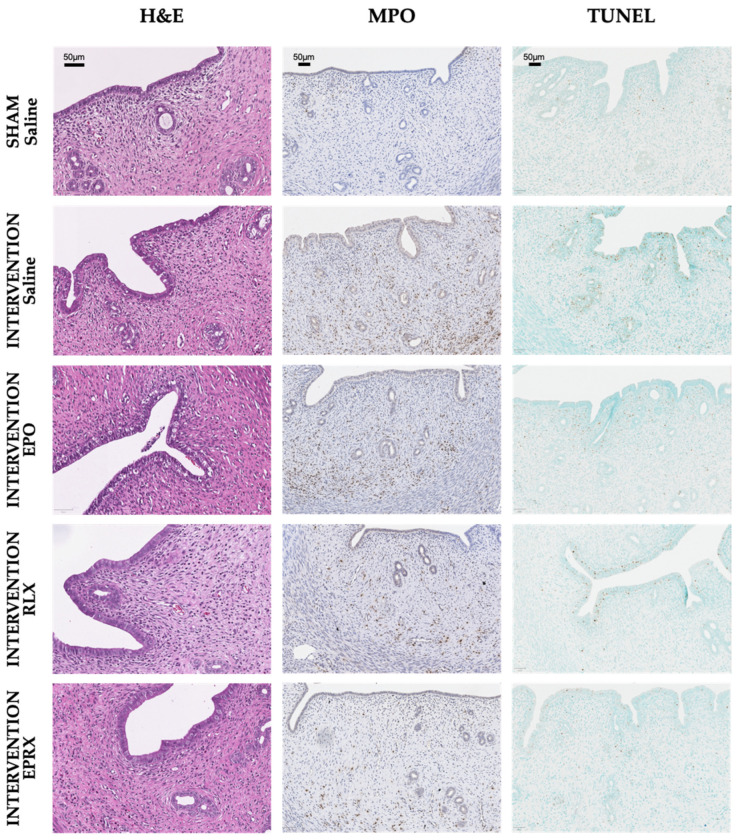
Micrographs of histological changes, MPO expression and TUNEL-positive cells in uterine tissue between sham + saline and all intervention groups. H&E—hematoxylin and eosin, MPO—myeloperoxidase, TUNEL—terminal deoxynucleotidyl transferase-mediated dUTP nick-end labeling EPO—erythropoietin, RLX—relaxin, EPRX—erythropoietin and relaxin.

Histological and immunohistochemical analysis revealed no significant differences between all sham-operated rats (Figure 1 and Figure 2). Samples of sham-operated rats presented with normal uterine tissue morphology—normally organized endometrial glands, only a mild infiltration with polymorphonuclear cells, with no vasoconstriction or hemorrhage. Contrarily, signs of severe uterine tissue architecture disruption were observed in all intervention groups; the highest histological score was evident in the intervention group pretreated with saline (6.0 (IQR 6.0; 7.3)). Pretreatment with RLX or RLX with EPO attenuated tissue damage, as samples in these groups presented with significantly lower histological scores (6.0 (IQR 6.0; 7.3) vs. 4.0 (IQR 3.0; 4.0) and 6.0 (IQR 6.0; 7.3) vs. 4.0 (IQR 3.0; 5.0), *p* = 0.017 and *p* = 0.038, respectively).

Myeloperoxidase (MPO) expression was the highest in the intervention + saline group (Figure 1 and Figure 2). The MPO-positive cell count was significantly lower in all intervention groups, after EPO, RLX or EPO with RLX application.

The intervention + saline group presented with the highest terminal deoxynucleotidyl transferase-mediated dUTP nick-end labeling (TUNEL)-positive cell number, which was significantly lower in the intervention + RLX and intervention + EPO and RLX groups (Figure 1 and Figure 2). EPO alone did not have any influence in lowering apoptotic cell numbers in the respective group compared to the intervention + saline group.

### 2.3. Gene Expression after Ischemia–Reperfusion Injury

**Figure 3:**
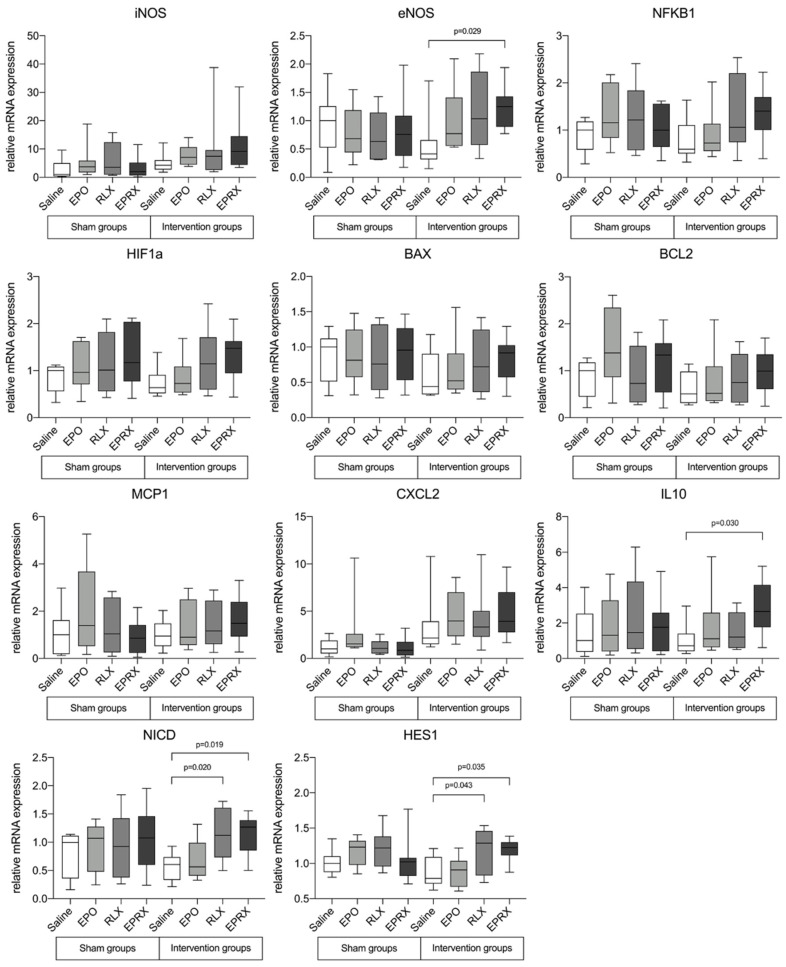
Quantitative RT-PCR of mRNA expression of iNOS, eNOS, NFKB1, HIF1A, BAX, BCL2, MCP1, CXCL2, IL10, NICD and HES1. Data were normalized to GAPDH gene expression. EPO—erythropoietin, RLX—relaxin, EPRX—erythropoietin with relaxin. Data are shown as median (Q1, Q3). The Kruskal–Wallis test was used in all cases.

Real-time quantitative polymerase chain reaction (qPCR) analysis revealed changes in several inflammatory and apoptosis genes between experimental groups in rat uterine tissue. No changes in the expression of iNOS, NFKB1, HIF1, BAX, BCL2, MCP1 and CXCL2 genes were detected after reperfusion in rat uterus tissue (Figure 3).

Pretreatment with RLX combined with EPO significantly upregulated the expression of eNOS, IL10, NICD and HES1 1.25- (*p* = 0.029), 2.65- (*p* = 0.030), 1.27- (*p* = 0.019) and 1.22-fold (*p* = 0.035), respectively. Furthermore, RLX alone resulted in significantly upregulated NICD and HES1 gene expression (1.12-fold, *p* = 0.020 and 1.29-fold, *p* = 0.043, respectively). Unfortunately, EPO alone had no significant impact on gene expression.

## 3. Discussion

Our study demonstrated that pretreatment with RLX or/and EPO significantly alleviates uterine tissue damage caused in an experimental IRI model. This is reflected by reduced lipid peroxidation, neutrophil infiltration, apoptosis, increased anti-inflammatory response and differential regulation of expression of genes involved in inflammation and apoptosis. In addition, pretreatment with RLX or RLX + EPO preserved uterine tissue morphology during IRI, which is important for further graft function.

IRI is a major factor limiting successful outcomes in solid organ transplantation [16]. It is defined as a multifactorial inflammatory condition involving hypoxia, metabolic stress, leukocyte extravasation, cellular death pathways and activation of the immune response [11].

Understanding these underlying mechanisms enables us to apply potential therapeutic approaches aimed at alleviating the deleterious effects of IRI. Currently, only several studies report the use of such agents to reduce IRI in the uterus. Sahin et al. report that the use of the calcineurin inhibitor Tacrolimus reduces oxidative stress in the uterus during IRI and significantly suppresses histopathological changes [17]. In a similar study, Sahin Ersoy et al. investigated another immunosuppressant, mycophenolate mofetil (MMF) [18]. This study determined that oxidative stress markers, such as 8-hydroxy-2′-deoxyguanosine (8-OHdG), MDA, MPO and ischemia-modified albumin, and cellular apoptosis were significantly attenuated in rats treated with MMF. Moreover, the use of MMF reduced IRI-induced uterus morphological changes. Comparable findings were reported by Aslan et al., who used oxytocin and kisspeptin premedication to significantly lower histological alterations and oxidative stress markers [19]. In essence, the same results were achieved by Atalay et al. by continuously administering remifentanil during ischemia–reperfusion [20]. Recently, the use of melatonin and glycine was shown to significantly reduce tissue MPO expression and increased SOD activity [21].

EPO is a glycoprotein hormone that stimulates erythropoiesis and is synthesized in the kidneys and fetal liver. In our study, it significantly decreased the number of MPO-positive cells in the uterus; however, a contradictive study by Tsompos et al. concluded that the use of EPO did not have any protective effects during uterus IRI [22]. This study mostly relied only on histological changes and no other IRI readouts were taken into account. Furthermore, their reported EPO dosage draws some suspicion, as it is extremely high and almost technically unachievable.

RLX is a peptide hormone that is being widely studied in transplantation research as a protective agent against IRI. It acts through various target molecules and pathways, some of them being organ-specific. RLX acts on leukocytes as it induces the expression of Notch1 in macrophages and reduces the expression of intracellular adhesion molecule 1, as well as neutrophil adhesion through the increased synthesis of nitric oxide [23,24]. The hepatoprotective mechanism of RLX is unique as it acts via glucocorticoid receptors and inhibits the release of cytochrome c from mitochondria, thus suppressing apoptosis [25]. Furthermore, RLX increases organ perfusion as it reduces vasoconstriction by inhibiting endothelin 1 production and increases vasodilation through the amplified production of nitric oxide [26]. With the qPCR analysis in our study, we confirmed that RLX acts through the Notch1 signaling and upregulates the expression of NICD and HES1. NICD further blocks NFkB, which is responsible for tissue inflammation and activates the HES1 pathway, blocking Caspase-3 and reducing cellular apoptosis [27].

qPCR analysis revealed another interesting result showing that the RLX and EPO combination upregulated eNOS gene expression. Inducible NOS expression was not affected by any of the experimental substances. Pretreatment with RLX alone showed only a tendency to upregulate eNOS gene expression, but this unfortunately did not reach significance. We could only speculate that the results were not significant due to the small sample size. These findings are partly in line with previously reported results of a study published by Bani et al. [28]. The synergistic effect of the RLX and EPO combination managed to significantly upregulate the expression of IL-10, which plays a central role in limiting the immune response [29]. Kageyama et al. reported that RLX alone managed to increase the expression of the IL-10 gene in liver tissue; however, there are no studies examining the effects of RLX and EPO combination on reducing IRI [23].

## 4. Materials and Methods

### 4.1. Animals

Eighty adult female (12-week-old) Sprague Dawley rats weighing 250–330 g were used in this experiment. All animals were obtained from Janvier Labs (Le Genest-Saint-Isle, France) and kept in the Division for Biomedical Research at the Medical University, Graz. An acclimatization period of 7 days before the experiments was applied for the animals under controlled environmental conditions on a 12 h light/dark cycle with room temperature set at 22 ± 1 °C and with access to fresh water and standard rat chow ad libitum. All experimental procedures and protocols were performed according to the 3R guidelines and were approved by the Austrian Ministry for Science, Research and Economy on the 17 September 2019 (Approval number: BMWF-66.010/0154-V/3b/2019).

### 4.2. Experimental Design

Before the experiments, rats were randomly assigned into eight groups (*n* = 10/group) and underwent either sham or experimental surgery. Each group was pretreated either with saline or 4000 IU/kg recombinant human erythropoietin (Erypo; Janssen-Cilag GmbH, Germany) or 5 μg/kg human relaxin-2 (Immundiagnostik AG, Bensheim, Germany), or the combination of RLX and EPO.

On the day of the experiment, animals were anesthetized in an anesthesia box using 2% 2 L/min isoflurane (Piramal-Isoflurane, Piramal Critical Care Deutschland GmbH, Hallbergmoos, Germany). After anesthesia induction, a mixture of anesthetic agents was injected intramuscularly, consisting of 0.15 g/kg medetomidine (Domitor, Orion Pharma, Vienna, Austria), 2 mg/kg midazolam (Midazolam, Accord Healthcare GmbH, Germany) and 5 μg/kg fentanyl (Fentanyl-Hameln, Hameln, Germany). During the experiment, rats were kept on a heating pad to maintain their body temperature at 37 °C, which was measured using a rectal probe. Heart rate and blood oxygen saturation were monitored constantly using a pulse oximeter device (Model 9847V; Nonin Medical BV, Amsterdam, The Netherlands), placed on one of the forelimbs. 

**Figure 4:**
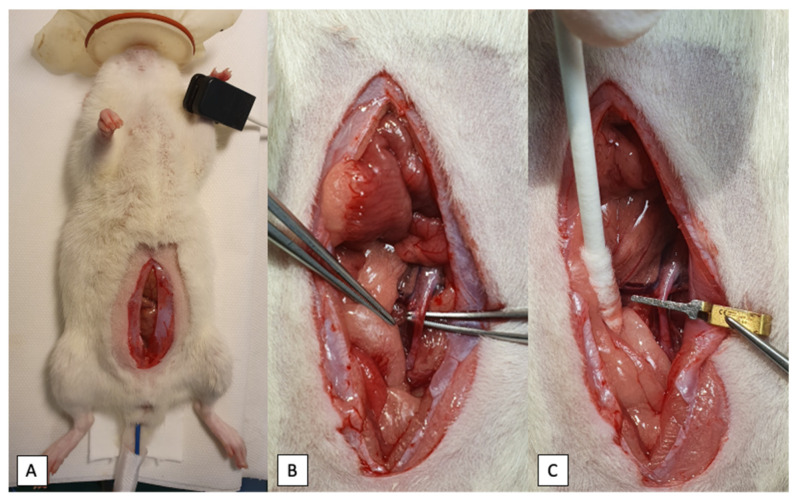
Surgical procedure for IR injury model. (**A**)—positioning of an animal prior to experiment, a 3–4 cm midline laparotomy. (**B**)—identification of the vessels, dissection of the aorta. (**C**)—occlusion of the aorta with microvascular bulldog clamp.

**Figure 5:**
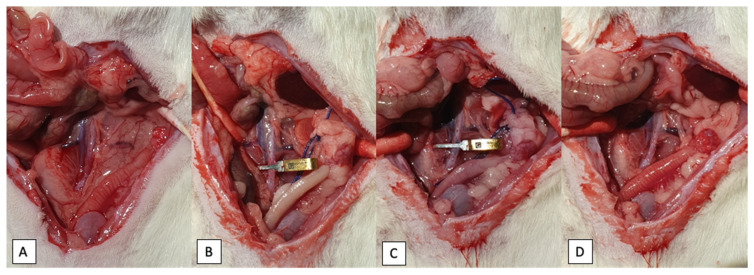
Blood flow changes in uterus during the experiment. (**A**)—before ischemia; (**B**)—start of ischemia; (**C**)—before reperfusion; (**D**)—start of reperfusion.

After the anesthesia had been achieved, a single injection of saline, EPO (4000 IU/kg), RLX (5 μg/kg) or EPO (4000 IU/kg) with RLX (5 μg/kg) resuspended in 200 μL of sterile saline was administered through the lateral tail vein using a 26 G needle (B.Braun, Melsungen, Germany). After the operation field was shaved and disinfected, a 3–4 cm midline laparotomy was performed and uterus, ovaries and relevant vessels were identified (Figure 4). Exactly 30 min after the injection of the respective substances, ischemia was induced by clamping the distal abdominal aorta approximately 1 cm above the bifurcation with a 20–25-g pressure microvascular bulldog clamp (Geister Medizintechnik GmbH, Tuttlingen, Germany). Ovarian arteries with surrounding fatty tissue were occluded bilaterally using surgical polyglactin 2 (Vicryl 2, Ethicon, Johnson & Johnson International) ligature. Sham-operated rats underwent the same dissection procedure except for the exclusion of the abovementioned vessels. After clamping the vessels, the cessation of blood flow was ascertained by observing a pale uterus color (Figure 5B). The abdominal wall was sutured continuously with polyglactin 4–0 suture and the animal was placed in a sideways position to ensure proper lung ventilation and oxygen supply. After 60 min of ischemia, reperfusion was initiated by releasing the clamp and sutures from the abdominal aorta and ovarian arteries bilaterally. By inspecting the uterus color, the restoration of blood flow was confirmed (Figure 5D). Reperfusion was maintained for 120 min. After this period, uterus and ovaries were procured and animals were euthanized by exsanguination. One uterus horn was immediately flash-frozen in liquid nitrogen and stored at −80 °C for biochemical analysis, and the other uterus horn was fixed in 4% formalin solution for further histological evaluation.

### 4.3. Tissue Morphological Evaluation

Uterine tissue was fixed in 4% formalin and embedded in paraffin after routine histological procedures were performed. Tissue sections of 2 μm were stained with hematoxylin and eosin and afterward evaluated by a blinded pathologist using an already published scoring system for uterus IRI [10] (Table 1). Uterus tissue score was calculated according to seven parameters seen under light microscopy.

### 4.4. Biochemical Analysis

Malondialdehyde (MDA) as a lipid peroxidation marker and antioxidant SOD activity were chosen for biochemical analysis. Frozen tissue samples were homogenized with ceramic beads in a phosphate-buffered solution or lysis buffer (catalog number: ab118970, Abcam) using the MagNA Lyser instrument (Roche Life Science. Mannheim, Germany) and centrifuged at 10,000 rpm for 10 min at 4 °C. The clear supernatant was collected and the following kits were used: Pierce™ BCA Protein Assay Kit (catalog number: 23225, Thermo Fisher Scientific, Waltham, MA, USA), Lipid Peroxidation (MDA) Assay Kit (catalog number: ab118970, Abcam), Superoxide Dismutase Colorimetric Activity Kit (catalog number: EIASODC, Invitrogen). All kits were utilized according to the manufacturer’s manual. Tissue MDA and SOD levels were measured and expressed per milligram (mg) of protein.

### 4.5. Immunohistochemical (IHC) Staining

Uterus tissue sections were stained with anti-MPO antibodies to evaluate tissue infiltration with neutrophils. After deparaffinization and antigen retrieval in a pressure cooker, 3% H_2_O_2_ solution in methanol was used to block the activity of endogenous peroxidase. Primary anti-MPO antibodies (catalog number: A0398, Dako, Glostrup, Denmark) were diluted 1:800 in Dako REAL Antibody Diluent (Dako, Glostrup, Denmark) and applied for 60 min at room temperature. The UltraVision LP Detection System: HRP Polymer (Thermo Fisher Scientific, Waltham, MA, USA) and DAB Chromogen (Dako, Via Real Carpinteria, CA, USA) were used to visualize the target antigen. Sections were counterstained with hematoxylin, scanned and evaluated. Anti-MPO positive cells were quantified as the percentage of positive cells among all cells using QuPath software (open-source software for Quantitative Pathology, version 0.2.0).

### 4.6. TUNEL Assay

Apoptotic cells were assessed using the TUNEL Assay Kit—HRP-DAB (catalog number: Ab206386, Abcam). The method was applied according to the manufacturer’s manual. Stained sections were scanned and positive cells were counted using QuPath software (open-source software for Quantitative Pathology, version 0.2.0).

### 4.7. Real-Time qPCR

Uterus tissue samples were snap-frozen and stored in liquid nitrogen until nucleic acid extraction. The tissue sample (weighing 50–100 mg) was homogenized in 1 mL TRIzol reagent (Invitrogen, Carlsbad, CA, USA) using a MagNA Lyser instrument (Roche Life Science, Mannheim, Germany) at 6500 rpm for 30 s. Nucleic acid was extracted according to the manufacturer’s protocol. RNA was quantified spectro-photometrically using the NanoDrop 2000 (Thermo Fisher Scientific, Waltham, MA, USA). Two micrograms of RNA were used for reverse transcription (High-Capacity cDNA RT Kit; Thermo Fisher Scientific, Waltham, MA, USA), according to the protocol provided by the manufacturer, in a final volume of 20 μL. Real-time PCR amplification and melting curve analysis were performed using a BioRad CFX96 TouchTM System (Bio-Rad Laboratories GmbH, Vienna, Austria). The amount of cDNA corresponding to an equivalent of 5 ng RNA was added to a reaction mix containing Promega GoTaq^®^ qPCR Master Mix (Promega, Madison, WI, USA), containing 1 μM of each primer in a final reaction volume of 10 μL. The PCR reaction mixture was subjected to an initial denaturation at 95 °C for 10 s, followed by 45 cycles of denaturation at 95 °C for 10 s, annealing at 58 °C for 20 s and elongation at 72 °C for 30 s, followed by a melting curve (60 to 95 °C). For detailed information on primers used, see Table 2. Gene expression was determined using Bio-Rad CFX Manager 3.1 (BioRad Laboratories GmbH, Vienna, Austria), using the Cq regression method embedded in the program. All PCR reactions were performed in duplicate, and relative gene expression was calculated by the 2^^−ddCT^ method, using GAPDH as a housekeeping gene.

### 4.8. Statistical Analysis

Statistical analysis was performed using the Statistical Package for the Social Sciences (SPSS) version 23 program (SPSS Inc., Chicago, IL, USA) and GraphPad Prism 9 (GraphPad Software, La Jolla, CA, USA). Distribution of variables was investigated using the Shapiro–Wilk test. Normally distributed data were analyzed by one-way ANOVA test. If any differences were observed, pairwise post-hoc Tukey’s test was used. Non-normally distributed data were evaluated by a Kruskal–Wallis test. When overall significant differences were observed, pairwise significance was tested using Dunn’s post-hoc test. Data are reported as median and quartiles (Q1; Q3). Differences were considered statistically significant when *p* < 0.05.

## 5. Conclusions

In conclusion, pretreatment with RLX, EPO or a combination of both EPO with RLX significantly alleviates uterine tissue damage caused by IRI. Combination of RLX with EPO was as effective as RLX alone, except that the combination of these substances yields an additional benefit for anti-inflammatory gene expression. Further studies are warranted before introducing these novel agents into clinical practice.

## Figures and Tables

**Table 1:** Light microscopy morphological scoring system for evaluation of the uterus IRI model.

Score	0	1	2
Inflammatory cells	Absent	Moderate amount of cells	Severe infiltration of cells
Vasoconstriction	Absent	Moderate < 20% small vessels	Severe > 20% small vessels
Hemorrhage	Absent	Subendometrial	Myometrial plus endometrial
Necrosis	Absent	<20% *	>20% *
Edema	Absent	<50% *	>50% *
Thrombosis	Absent	<50% of the vessels	>50% of the vessels
Endometrial loss of cells	Absent	<20% *	>20% *

* Percentages are calculated as (surface of the affected area/surface of the whole section) × 100.

**Table 2:** Genes used for quantification and primer information.

Acc. Number	Forward Primer (5′–3′)	Reverse Primer (5′–3′)	Product Length
***GAPDH—Glyceraldehyde-3-Phosphate Dehydrogenase***			
NM_017008.4	AGTGCCAGCCTCGTCTCATA	GGTAACCAGGCGTCCGATAC	77 bp
***eNOS—Endothelial Nitric Oxide Synthase***			
NM_021838.2	ATTGGCATGAGGGACCTGTG	CCGGGTGTCTAGATCCATGC	81 bp
***iNOS—Inducible Nitric Oxide Synthase***			
NM_012611.3	TTGGTGAGGGGACTGGACTTT	CCGTGGGGCTTGTAGTTGA	86 bp
***HIF1a—Hypoxia Inducible Factor 1 Subunit Alpha***			
NM_024359.1	CGGCGAGAACGAGAAGAAAA	ACTCTTTGCTTCGCCGAGAT	89 bp
***BAX—BCL2-Associated X Protein***			
NM_017059.2	GACACCTGAGCTGACCTTGG	AGTTCATCGCCAATTCGCCT	87 bp
***BCL2—B-Cell Lymphoma 2***			
NM_016993.1	GACTGAGTACCTGAACCGGC	GCATGCTGGGGCCATATAGT	89 bp
***MCP1—Monocyte Chemoattractant Protein-1***			
NM_031530.1	CTTCCTCCACCACTATGCAGG	GATGCTACAGGCAGCAACTG	71 bp
***NFKB1—Nuclear Factor of Kappa Light Polypeptide Gene Enhancer in B-Cells***			
NM_001276711.1	GGACAACTATGAGGTCTCTGGG	TTCAATGGCCTCTGTGTAGCC	82 bp
***NICD—Notch Intracellular Domain***			
NM_001105721.1	CGGGACATCACGGATCACAT	ATTCATCCAAAAGCCGCACG	88 bp
***HES1—Hairy And Enhancer Of Split 1***			
NM_024360.3	ATGACAGTGAAGCACCTCCG	GGTACTTCCCCAACACGCTC	85 bp
***IL10—Interleukin 10***			
NM_012854.2	ACCCGGCATCTACTGGACT	GTTTTCCAAGGAGTTGCTCCC	89 bp
***CXCL2—C-X-C Motif Chemokine Ligand 2***			
NM_053647.1	CTGACTACACCACCTCCACAC	GCCTTGAAAGCCCTCTGACT	87 bp

## Data Availability

All data relevant to the study are included in the article.
